# Monocytes strongly induce (myo)fibroblast contraction in a 3D skin model to understand inflammation-fibrosis crosstalk

**DOI:** 10.1038/s41598-026-62996-0

**Published:** 2026-07-19

**Authors:** D. C. Zanin-Silva, N. J. T. van Kooten, T. I. Papadimitriou, D. N. Dorst, B. Walgreen, E. L. Vitters, M. H. J. van den Bosch, M. I. Koenders, A. P. M. van Caam

**Affiliations:** 1 Basic and Applied Immunology Graduate Program, Ribeirão Preto Medical School, University of São Paulo, Ribeirão Preto, Brazil; 2 Experimental Rheumatology, Department of Rheumatology, Radboud University Medical Centre, Nijmegen, The Netherlands

**Keywords:** Monocytes, Myofibroblasts, Fibrosis, Systemic sclerosis, 3D in vitro models, Skin, Cell biology, Diseases, Immunology, Rheumatology

## Abstract

Systemic sclerosis (SSc) is an autoimmune disease characterized by excessive fibrosis and tissue stiffness, in which monocytes and macrophages are increasingly recognized as key contributors to pro-fibrotic myofibroblast formation, although the underlying mechanisms remain incompletely understood. Here, we used a three-dimensional (3D) skin model to study how CD14^+^ monocytes, M1 and M2-like macrophages induce fibroblasts activation and contraction in collagen type I hydrogels. We identified that the co-culture of dermal (myo)fibroblasts with monocytes displayed strong spontaneous hydrogel contraction, coupled with an upregulation of myofibroblast activation-associated markers, such as alpha-smooth muscle actin (α-SMA). Using transcription factor-responsive reporter constructs and small-molecule inhibitors, we demonstrated that monocytes-(myo)fibroblasts communication was mediated by JAK/STAT3 and TGF-β/Smad2/3 signaling pathways. Flow cytometry analyses revealed that monocytes, after interacting with (myo)fibroblasts, differentiated into a mixed M1/M2 polarization phenotype, characterized by CD163, CD206, CD86, and HLA-DR expression. Both M1 and M2-like macrophages promoted significant (myo)fibroblast contraction, which could be mimicked by supernatant transfer. TGF-β neutralization but not IL-6 blocking abolished this effect. This study demonstrates that monocytes/macrophages can strongly induce (myo)fibroblasts activation/contraction. Together, our work contributes to elucidating pathways and mechanisms associated with skin fibrosis in SSc and paves the way for developing new platforms for targeted therapy testing.

**Supplementary Information:**

The online version contains supplementary material available at https://doi.org/10.1038/s41598-026-62996-0.

## Introduction

Systemic sclerosis (SSc) is a severe autoimmune disease characterized by vasculopathy, immunological dysregulation, and fibrosis of the skin and internal organs^1^. The immune response plays a central role in the establishment and maintenance of SSc fibrosis, by promoting the differentiation, activation, and survival of myofibroblasts^2^. Myofibroblasts are a subset of fibroblasts distinguished by their stress fibers, i.e. intracellular structures made of actin and myosin, that give them contractile properties, and their ability to produce high amounts of extracellular matrix (ECM) components, such as collagen type 1, type 3, and fibronectin. Notably, myofibroblasts express alpha-smooth muscle actin (α-SMA), which is associated with increased skin stiffness in SSc^3,4^.

Various immune cells, such as macrophages and their precursing monocytes, are implicated with myofibroblasts formation during fibrotic conditions^5,6^. In SSc, monocyte/macrophage infiltration is seen across different affected organs, such as the lungs and the skin^7,8^. Recently, spatial transcriptomics on SSc patients’ skin biopsies revealed fibrotic niches enriched with fibroblasts and macrophages, closely linked to disease severity^9^. Moreover, biophysical changes in circulating monocytes from SSc patients, including alterations in cell morphology (e.g., cell area as a measure of cell size) and deformability, reflect a pathological activation state and are associated with disease activity, microvascular damage, and fibrosis extent^10^.

Nonetheless, the mechanisms by which monocytes/macrophages induce myofibroblast activation and contraction in SSc remain incompletely understood.

Experimental models exploring mechanisms related to myofibroblast activation and contraction often incorporate key biomechanical properties of the fibrotic microenvironment in which these cells reside^11–13^. Collagen hydrogels present a valuable solution for this purpose, as they contain a component naturally produced by myofibroblasts, mimicking characteristics of a fibrotic ECM^14^. Within such 3D systems, cell co-cultures are essential to capture paracrine signaling and direct cell-cell interactions that regulate myofibroblast behavior^11,13^. However, few current in vitro models of SSc models incorporate immune-stromal cell interactions^11,13,15^.

Here we used a three-dimensional (3D) skin model to understand whether monocytes/macrophages could cause (myo)fibroblasts activation and contractile activity in collagen type 1 hydrogels. We recently demonstrated a significant role for CD8^+^ T lymphocytes in inducing spontaneous myofibroblasts contraction in these hydrogels^15^. However, it has not yet been elucidated whether other immune cells also implicated in the inflammation-fibrosis pathological axis in SSc, such as monocytes/macrophages, could exhibit a similar effect.

## Results

### Monocytes strongly induce (myo)fibroblast contraction in 3D collagen hydrogels

To investigate the potential role of monocytes in driving (myo)fibroblasts contractile activity in connective tissue diseases like SSc, we co-cultured human primary dermal (myo)fibroblasts, obtained from a healthy individual, with CD14^+^ monocytes isolated from healthy peripheral blood mononuclear cells (PBMCs) in 3D type I collagen hydrogels.

**Fig. 1:**
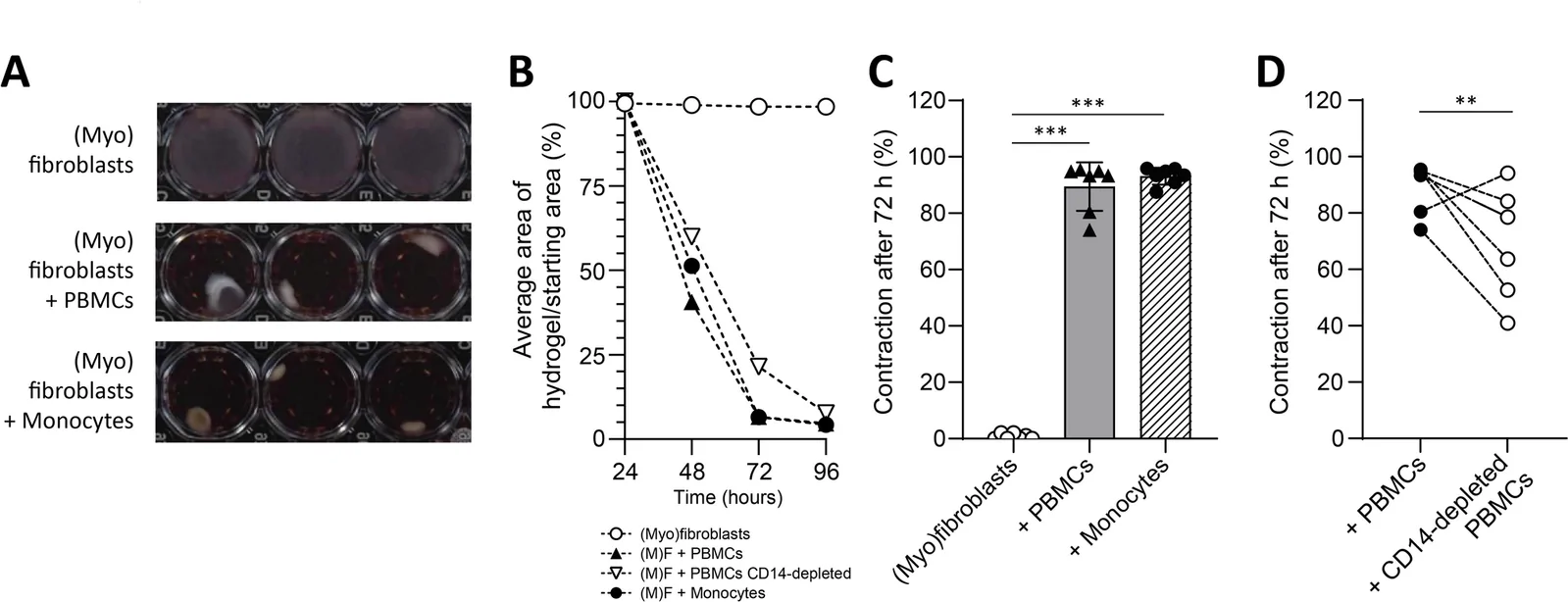
Monocytes strongly induce (myo)fibroblast contraction in the 3D skin collagen hydrogel model. (**A**) Representative scanned pictures (after 72 h) of uncontracted hydrogels containing only (myo)fibroblasts or in co-culture with immune cells (PBMCs or monocytes). (Myo)fibroblast contraction is seen by a reduction in the surface area of the hydrogels. (**B**) Representative kinetics of (myo)fibroblast contractile activity over time (from one PBMC donor). Hydrogels with only (myo)fibroblasts did not contract. Inversely, hydrogels, in which (myo)fibroblasts were co-cultured with monocytes or PBMCs, displayed a robust contraction starting after 48 h, reaching its maximum at 72 h. (**C**) Contraction was evaluated by the average surface area of a contracted hydrogel group in triplicate divided by the total area of an uncontracted hydrogel. Co-culture of (myo)fibroblasts with monocytes or (myo)fibroblasts with PBMCs resulted in contraction of similar strength. (**D**) Depletion of CD14 + cells from PBMCs profoundly impaired hydrogels’ contraction. Each shape represents the average of 3 technical replicates of one PBMC donor, *n* = 7. Groups were statistically compared with one-way ANOVA with Tukey’s multiple comparisons test (**p < 0.01, ***p < 0.001).

Hydrogels containing only (myo)fibroblasts did not contract (Fig. 1a) over 96 h (h) study (**Fig. b**), neither do hydrogels with only monocytes (data not shown). On the other hand, strong spontaneous contraction was observed in all hydrogels in which (myo)fibroblasts were co-cultured with monocytes, starting after 48 h of the experiment (**Fig. b**). Similarly, co-culture of (myo)fibroblasts with PBMCs instead of monocytes, resulted in contraction of similar strength and comparable speed (Fig. 1b, c), equivalent to what we observed previously^15^. To test the relative contribution of monocytes to PBMCs-induced contraction, we depleted CD14^+^ cells before co-culturing with (myo)fibroblasts and observed that this significantly impaired hydrogels contraction (Fig. 1d), indicating that monocytes clearly promote (myo)fibroblast contractile activity.

### Monocytes lead to activation markers expression in (myo)fibroblasts

**Fig. 2:**
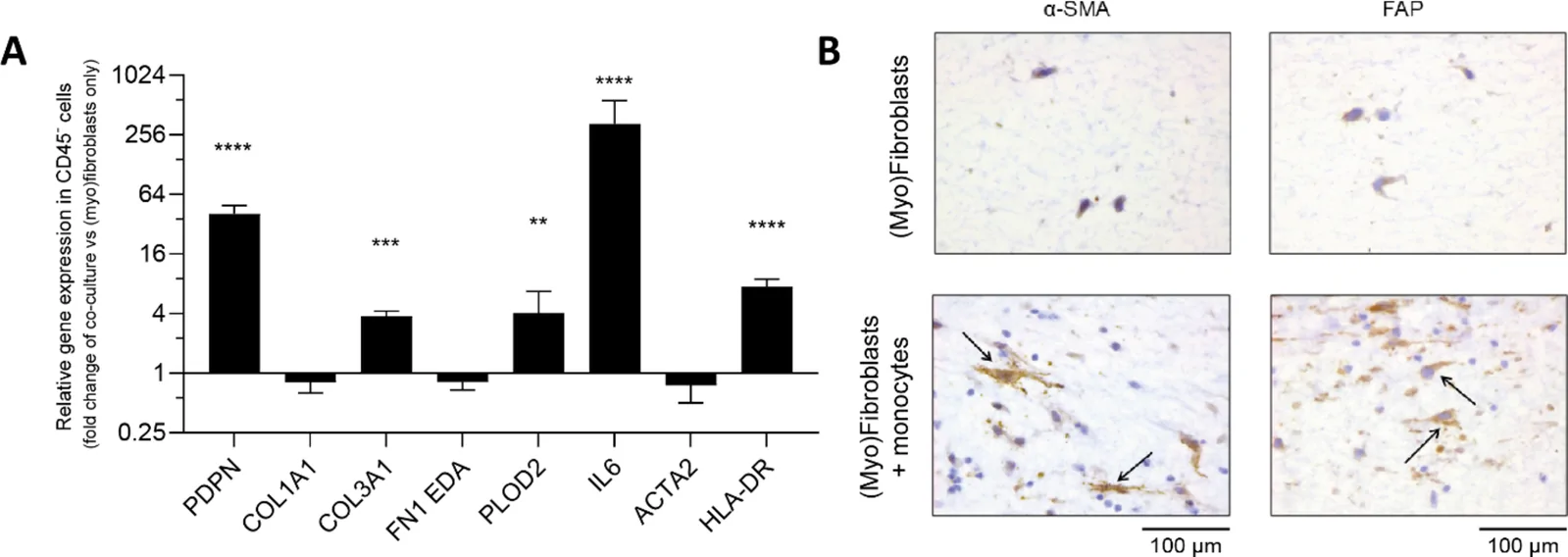
Monocytes promote robust expression of activation markers in (myo)fibroblasts. (**A**) Sorted CD45- (myo)fibroblasts were analyzed for expression of pro-fibrotic, inflammatory, and activation-associated genes after 24 h (h) co-culture with monocytes in collagen hydrogels. Values (mean ± SEM) indicate relative gene expression (2^−ΔΔCt^) measured with qPCR. Reference genes used were GAPDH and RPS27A. Analyses were done by two-way ANOVA with Sidak’s multiple comparisons test (**p < 0.01, ***p < 0.001, ****p < 0.0001). (**B**) Representative immunohistochemistry staining, performed at 72 h, showing robust expression of α-SMA (alpha-smooth muscle actin) (lower left) and fibroblast activation protein (FAP) (lower right) in the hydrogels containing (myo)fibroblasts co-cultured with monocytes compared to those with only (myo)fibroblasts (top panels). The small blue nuclei in the bottom panels are monocytes. (Myo)fibroblasts are clearly positive for α-SMA and FAP (black arrows), with a typically elongated shape. The immunohistochemistry images shown are representative of a single PBMC donor, with similar staining patterns consistently observed across all donors (*n* = 3) analyzed.

To study whether and how monocytes also influence (myo)fibroblasts gene and protein expression, we co-cultured both cell types in 3D hydrogels and measured pro-fibrotic, inflammatory, and activation-associated markers, such as alpha smooth muscle actin (α-SMA), fibroblast activation protein (FAP), and type I collagen^16,17^ by quantitative real-time PCR. To assess (myo)fibroblast-specific gene expression, CD45⁺ cells were depleted using magnetic beads, resulting in a negatively selected CD45⁻ (myo)fibroblasts-enriched population. 24 h (h) after co-culture with monocytes, (myo)fibroblasts exhibited a significant increase in gene expression of podoplanin (*PDPN*), *COL3A1*, *HLA-DR*, Procollagen-Lysine,2-Oxoglutarate 5-Dioxygenase 2 (*PLOD2*), and interleukin-6 (*IL-6*) (Fig. 2a), reflecting an activated and pro-inflammatory state.

Subsequently, we confirmed this monocyte-induced activated phenotype at protein level by immunohistochemical staining of the hydrogels, performed 72 h after establishing the co-culture. (Myo)fibroblasts co-cultured with monocytes were clearly positive (indicated by arrows) for fibroblast activation protein (FAP) and alpha-smooth muscle actin (α-SMA), indicating strong activation by monocytes (Fig. 2b).

### Monocyte-induced JAK/STAT and TGF-β/Smad signaling pathways in (myo)fibroblasts during co-culture

To elucidate the mechanisms underlying (myo)fibroblasts activation, we analyzed which intracellular signaling pathways are triggered in response to monocytes. For this, we used dermal (myo)fibroblasts transfected with transcription factor-responsive reporter constructs^18^(**Supplementary Fig. 1**) including Smad-binding elements (SBE) to capture canonical TGF-β signaling; STAT-inducible elements (SIE) reflecting JAK/STAT activation; NF-κB response elements, representing inflammatory signaling; interferon-stimulated response elements (ISRE), indicative of type I interferon responses; nuclear factor of activated T-cells 5 (NFAT-5) response elements associated with osmotic stress signaling, and cyclic adenosine monophosphate (cAMP) response elements (CRE), reflecting cAMP/PKA pathway activation.

**Fig. 3:**
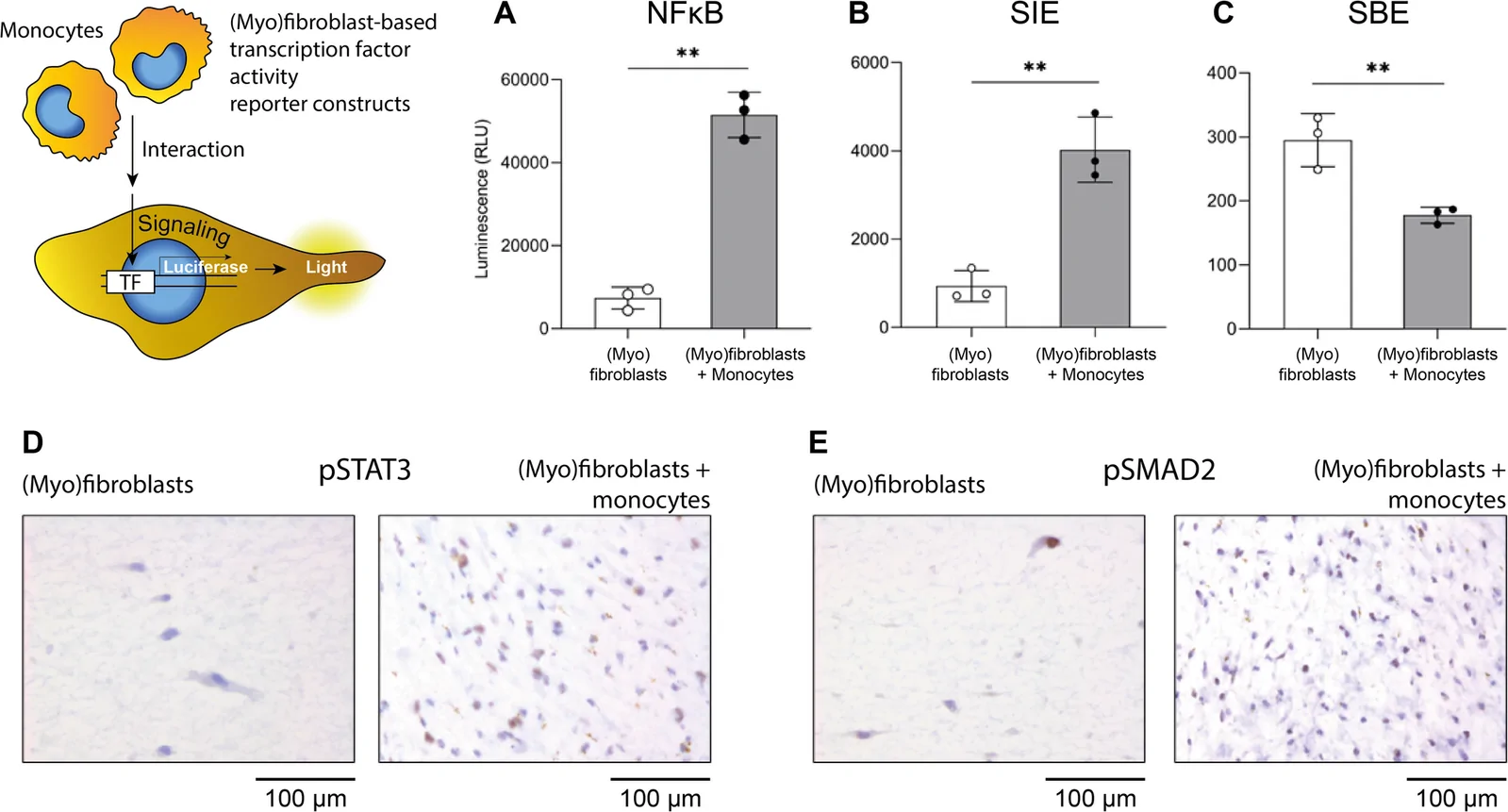
Signaling pathways activated in (myo)fibroblasts during the co-culture with monocytes. Dermal (myo)fibroblasts transfected with transcription factor-responsive reporter constructs were used to monitor the activation of signaling pathways. Following stimulation (by co-culture with monocytes), transcriptional activation drives luciferase expression, which is subsequently quantified by luminescence as an indirect measure of pathway activity. Luminescence values were measured in (myo)fibroblasts transfected with transcription factor-responsive reporter constructs NF-κB (**A**), SIE (**B**), and SBE (**C**) in the presence of monocytes (for 24 h) in the collagen hydrogels. Hydrogels with only (myo)fibroblasts (without any stimulation) were used as negative controls. Statistical analyses were performed using Student’s t-test (**p < 0.01). Each dot represents the average of 3 technical replicates of one PBMC donor, *n* = 3. The phosphorylation of STAT3 (pSTAT3) (**D**) and Smad2 (pSmad2) (**E**) was clearly observed by immunohistochemistry in monocyte-containing hydrogels (right) when compared to (myo)fibroblasts cultured alone (left), demonstrating induction of TGF-β and STAT3 signaling in the co-culture. The immunohistochemistry images shown are representative of a single PBMC donor, with similar staining patterns consistently observed across all donors (*n* = 3) analyzed. NF-κB: Nuclear factor-κB response element; SIE: Interleukin (IL)-6 sis-inducible element or STAT3 response element, and SBE: SMAD binding element.

After 24 h of co-culture with monocytes, the transcriptional activity of the NF-κB (Fig. 3a) and SIE (Fig. 3b) constructs was increased, whereas SBE (Fig. 3c) and ISRE (**Supp.** Figure 2a) activity was diminished. NFAT-5 and CRE transcriptional activity remained unchanged after the co-culture (**Supp** Fig. 2b, c), demonstrating no evidence for the involvement of osmotic stress-related or cAMP/PKA-dependent pathways triggered by monocytes in (myo)fibroblasts in our model.

The induction of the JAK/STAT signaling pathway by monocytes in (myo)fibroblasts was subsequently confirmed by immunohistochemistry, as evidenced by a clear phosphorylation of STAT3 (pSTAT3) in the hydrogels (Fig. 3d). In the hydrogels containing only (myo)fibroblasts, pSTAT3 staining was not detected (Fig. 3d). Additionally, the unexpected results showing reduced SBE (Smad binding element) luciferase activity (Fig. 3d) led us to further investigate the activation state of other components associated with this pathway. In contrast to the reduced SBE transcriptional activity, immunohistochemistry analysis revealed strong Smad2 phosphorylation in (myo)fibroblasts during the co-culture with monocytes (Fig. 3f), indicating that Smad signaling activation at the receptor level (e.g., Smad phosphorylation following TGF-β receptor engagement) does not directly translate into increased gene expression, likely due to additional regulatory mechanisms, such as post-translational changes^19^. Together, these results suggest that the JAK/STAT and TGF-β/Smad pathways orchestrate monocyte-induced (myo)fibroblasts activation in our 3D co-culture model.

### Essential role for JAK/STAT and TGF-β/Smad signaling in (myo)fibroblast contractile activity induced by monocytes

To determine whether the activation of the observed pathways was also involved with the (myo)fibroblasts contraction, we treated the co-culture hydrogels with different pharmacological inhibitors. For this, we used tofacitinib, a JAK/STAT signaling inhibitor^20^ and/or SB-505,124, a selective inhibitor targeting the type 1 TGF-β receptor (TGFBR1), and thus Smad signaling^21^. We also tested two other compounds known to block other potential relevant signaling pathways: IKK-16, a selective inhibitor of IκB kinase (IKK)^22^, and TAK-242, a toll-like receptor 4 (TLR4) signaling inhibitor^23^.

**Fig. 4:**
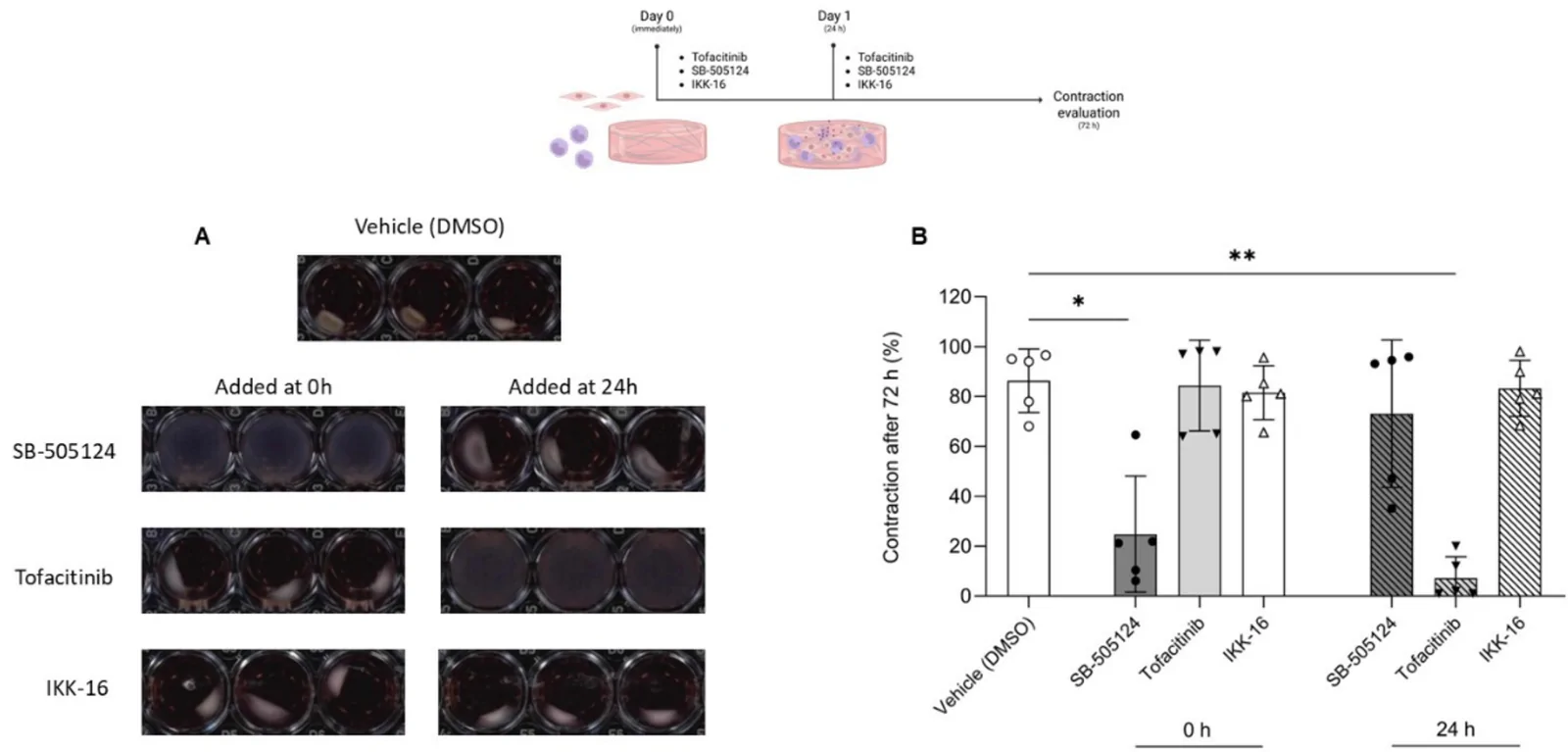
TGF-β/Smad and JAK/STAT signaling pathways are important regulators in monocyte-driven (myo)fibroblast contraction. The co-culture of (myo)fibroblasts and monocytes was treated with SB-505,124 (5 µM), tofacitinib (1 µM), or IKK-16 (1,5 mM) either immediately (0 h) after hydrogels generation or 24 h later, and subsequent contraction (at 72 h) was compared with vehicle-treated (DMSO) (control). (**A**) Representative scanned images of the co-cultured hydrogels treated with SB-505,124, tofacitinib or IKK-16 at the different mentioned time points (0–24 h). (**B**) Hydrogels treated immediately (0 h) with SB-505,124 displayed impaired contraction, which did not occur when the inhibitor was added later (at 24 h). Inversely, hydrogels receiving tofacitinib at 0 h showed strong spontaneous contraction, which did not happen upon treatment after 24 h. Blocking the NF-κB pathway did not affect (myo)fibroblasts contractile activity at any time point (0–24 h). Each shape represents the average of 3 technical replicates of one PBMC donor, *n* = 5. Groups were statistically compared with one-way ANOVA followed by Tukey’s multiple comparisons test (*p < 0.05, **p < 0.01).

3D hydrogels containing (myo)fibroblasts plus monocytes treated with SB-505,124 from the start of the co-culture exhibited a significant reduction in contraction after 72 h compared to those treated with only vehicle DMSO (Fig. 4). However, when SB-505,124 was added 24 h (h) after hydrogels’ preparation, no inhibition of contraction was observed (Fig. 4), suggesting that early activation of TGF-β signaling is a critical step in the model. In contrast, hydrogels receiving NF-κB inhibitor, IKK-16, at either time point (0–24 h) displayed no impairment in (myo)fibroblasts contraction (Fig. 4), indicating that this signaling pathway is not a driver of the observed contractile phenotype. Similarly, blocking TLR4 signaling using TAK-242 at the start of co-culture (0 h) did not affect (myo)fibroblasts contractile activity (**Supp** Fig. 3), suggesting that damage-associated molecular patterns (DAMPs) signaling is also not involved in this context. Interestingly, tofacitinib strongly inhibited hydrogel contraction when added after 24 h rather than at the start of the experiments (Fig. 4), which suggests that JAK/STAT signaling is activated or only relevant at a later stage. Overall, the use of pharmacological inhibitors allowed us to identify JAK/STAT and TGF-β/Smad as key signaling pathways involved in monocyte-induced (myo)fibroblast contraction in our 3D skin model.

### Interaction with (myo)fibroblasts leads monocytes to differentiate into macrophages with a mixed M1/M2-like phenotype

**Fig. 5:**
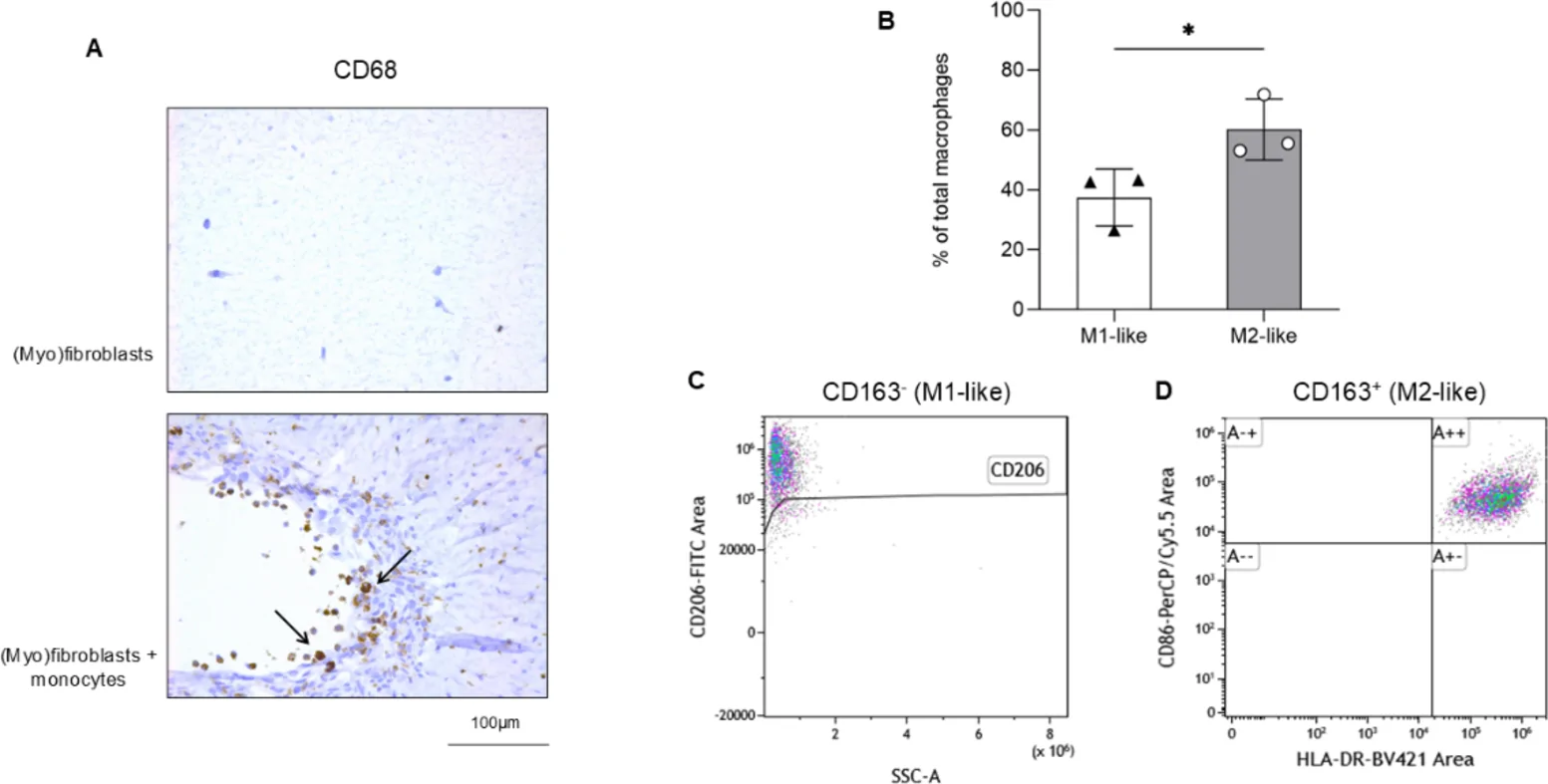
Interaction with (myo)fibroblasts promotes monocyte-to-macrophage differentiation. (**A**) Representative immunohistochemistry staining showing CD68 expression in monocyte-containing hydrogels (down). CD68^+^ cells (brown staining) display a rounded morphology and are indicated by black arrows. These cells are observed in close proximity to CD68^−^ spindle-shaped (myo)fibroblasts within the 3D collagen matrix. The immunohistochemistry images shown are representative of a single PBMC donor, with similar staining patterns consistently observed across all donors (*n* = 3) analyzed. (**B**) Flow cytometry analyses showing the percentage of total macrophages (CD45^+^CD68^+^) isolated from the hydrogels after enzymatic digestion and subsequently CD45^+^ magnetic selection. Cells (CD45^+^CD68^+^) were classified into M1-like (CD163^−^) or M2-like (CD163^+^) macrophages. Comparisons were performed using Student’s t-test (*p < 0.05). Each shape represents the average of 3 technical replicates of 1 PBMC donor, *n* = 3. (**C**,** D**) Interaction with (myo)fibroblasts drives monocytes toward M1-M2 mixed macrophage polarization phenotype. Representative flow cytometry plots of macrophage subsets isolated from collagen hydrogels co-cultured with (myo)fibroblasts and monocytes. (**C**) M1-like macrophages (CD163⁻) were gated for CD86⁺HLA-DR⁺ but also exhibited detectable CD206 expression. (**D**) M2-like macrophages (CD163⁺) expressed canonical M2 markers (CD206⁺) while also showing M1-associated markers (CD86⁺HLA-DR⁺).

We further examined how direct interaction with the (myo)fibroblasts within the hydrogels shaped monocyte differentiation. Once in the tissue, monocytes can differentiate into macrophages, and CD68, a type I transmembrane glycosylated protein, is the molecule commonly used as a marker to identify these cells in human tissues^24,25^. Examining the histology of our co-cultured hydrogels, we identified clear CD68 expression after 72 h of the experiment, suggesting monocyte-to-macrophage differentiation (Fig. 5a). Interestingly, we noted that many of the CD68^+^ cells were distributed forming a sort of “surrounding layer” covering the (myo)fibroblasts in the hydrogels, a phenomenon also seen during wound healing processes^26,27^.

After enzymatic digestion of the hydrogels, the CD45^+^ cell fraction was selected and stained for flow cytometry analysis to assess their immunophenotypic profile. We identified macrophages based on the expression of CD68 (CD45^+^CD68^+^) and subsequently classified them as M1- or M2-like macrophages (the gating strategy is detailed in **Supp** Fig. 4) based on the absence or presence of scavenger receptor CD163, respectively^28,29^.

Our analyses demonstrated that co-culture with (myo)fibroblasts led to the appearance of a significant percentage of M2-like macrophages (CD45^+^CD68^+^CD163^+^) (Fig. 5b) compared to M1-like cells (CD45^+^CD68^+^CD163^−^). As expected, M2-like macrophages were positive for the expression of CD206 (mannose receptor) (**Supp** Fig. 4), a classical marker of M2 profile^30,31^. Moreover, we observed that M1-like macrophages, in addition to the expression of markers traditionally associated with their activation state, such as CD86 and HLA-DR^29^, also expressed CD206 (Fig. 5c). In a similar manner, M2-like cells expressed markers associated with M1 polarization (Fig. 5d), HLA-DR and CD86, suggesting that interaction with (myo)fibroblasts induces monocytes to differentiate into macrophages with a mixed M1/M2 polarization phenotype, which may reflect a contribution to both pro-inflammatory and pro-fibrotic processes.

### In vitro-generated M1- and M2-like macrophages promote (myo)fibroblasts activation and contractile activity

Finally, we aimed to verify whether polarized M1 or M2 macrophages could also induce (myo)fibroblasts activation and contractile activity. Although classically activated M1 macrophages are known for their pro-inflammatory role, they are also described as important players during fibrotic conditions, including in the lungs^32^ and peritoneum^33^. On the other hand, M2 macrophages are primarily associated with tissue repair and regeneration and are more commonly linked to fibrosis^34,35^. To verify each cell subset’s influence, we produced hydrogels containing (myo)fibroblasts and macrophages derived from monocytes, which were polarized in vitro using a cytokine cocktail (see Methods) into the two well-known activation states: GM-CSF-generated M1 or M-CSF-generated M2.

**Fig. 6:**
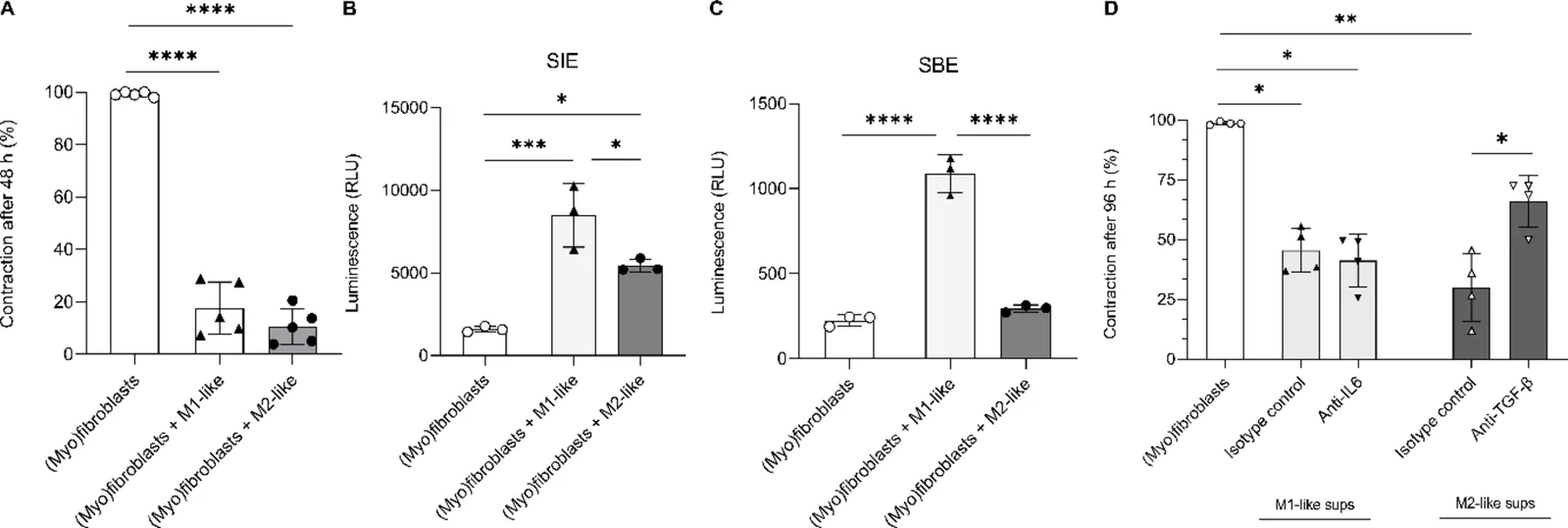
M2-like macrophages induce (myo)fibroblasts contraction through TGF-β in the hydrogels. (**A**) Hydrogels containing co-culture of (myo)fibroblasts and in vitro-generated M1- or M2-like macrophages showed a strong spontaneous contraction, reaching its maximum at 48 h (not shown). Hydrogels with only (myo)fibroblasts did not contract. Each shape represents the average of 3 technical replicates of one PBMC donor, *n* = 5. Groups were statistically compared with one-way ANOVA and Tukey’s multiple comparisons test (****p < 0.0001). (**B**, **C**) To assess signaling pathway activation, luminescence values were measured in (myo)fibroblasts transfected with SIE (**B**) and SBE (**C**) transcription factor-responsive reporter constructs in the presence (for 24 h) of M1- or M2-like macrophages in the collagen hydrogels. Hydrogels with only (myo)fibroblasts were used as negative controls. Statistical analyses were performed using Student’s t-test (*p < 0.05, ***p < 0.001, ****p < 0.0001). Each shape represents the average of 3 technical replicates of 1 PBMC donor, *n* = 3. (**D**) M1 or M2-like macrophage culture supernatants were incubated for 1 h with neutralizing antibodies against IL-6 or TGF-β, respectively. An IgG isotype antibody was added as a control. Blocking TGF-β significantly impaired M2-like supernatant-induced contraction. Differently, IL-6 neutralization in the M1-like supernatant had no effect on preventing (myo)fibroblasts contraction. Each shape represents the average of 3 technical replicates of 1 PBMC donor, *n* = 4. Groups were statistically compared using one-way ANOVA with Tukey’s multiple comparisons test (*p < 0.05, **p < 0.01).

We observed that after two days (48 h) after hydrogels’ preparation, both M1- and M2-like cells were strongly capable of inducing (myo)fibroblasts contractile activity (Fig. 6a). Additionally, using (myo)fibroblasts transfected with transcription factor-responsive reporter constructs, we observed that M1-like macrophages induced significant SIE transcriptional activity, more than M2-like cells (Fig. 6b), indicating that M1-like macrophages have a greater capacity to stimulate inflammatory signaling in (myo)fibroblasts. Moreover, (myo)fibroblasts also exhibited increased SBE activity in the presence of M1 (Fig. 6c) compared to M2-like cells. Increased transcriptional activity was further observed for the NF-kB and ISRE (**Supp** Fig. 5a, b), whereas no changes were detected in the CRE and NFAT-5 cells (**Supp** Fig. 5c, d).

To assess whether the effect of macrophage-induced (myo)fibroblasts contraction could be attributed to released soluble mediators, we used the culture supernatant from the M1- or M2-like macrophages to stimulate collagen hydrogels containing only (myo)fibroblasts. Although hydrogels receiving either M1 or M2-like supernatant both exhibited strong spontaneous contraction after 96 h of culture (Fig. 6d), this response was less pronounced than with the direct co-culture, suggesting that cell-cell contact plays a significant role in promoting (myo)fibroblast contraction.

Finally, we aimed to link the results of the enhanced SIE (JAK/STAT) and SBE (SMAD) signaling pathways to the role of soluble mediators, IL-6 and TGF-β, in driving (myo)fibroblasts contraction. For this, we incubated M1-like supernatants with anti-IL-6 and M2-like supernatants with anti-TGF-β neutralizing antibodies, based on the predominant cytokine profile of each macrophage subtype, and then analyzed if these cytokines mediated (myo)fibroblast contraction. Hydrogels treated with M2-like supernatant incubated with TGF-β neutralizing antibody displayed a significant reduction in the contraction process compared to those treated with an isotype control antibody (Fig. 6d), supporting a role for M2-like cell-derived TGF-β in driving (myo)fibroblasts contractile activity. On the other hand, IL-6 neutralization in the M1-like supernatant had no effect on preventing contraction of the hydrogels (Fig. 6d), pointing to the involvement of additional M1-like cell-derived mediators in this process.

## Discussion

During homeostasis, myofibroblasts play an essential role in tissue remodeling and wound healing process^36^. However, their sustained activation can substantially contribute to the development of various fibrotic diseases, such as systemic sclerosis (SSc)^4^ and Dupuytren’s contracture^37^. Here, we employed a three-dimensional (3D) skin collagen type 1 hydrogel model consisting of a co-culture of both healthy primary dermal (myo)fibroblasts and monocytes/macrophages, enabling the assessment of immune cell-driven (myo)fibroblasts activation and contractile activity, which reflects increased skin stiffness and fibrosis in SSc. Through the co-culture, we observed a crucial role of monocytes/macrophages in these processes.

Enhanced (myo)fibroblasts contractility was accompanied by upregulation of certain genes such as PDPN, COL3A1, HLA-DR, PLOD2, and IL-6, reflecting an activated and pro-inflammatory phenotype. These genes have been also found elevated in SSc patients’ skin myofibroblasts, coupled with increased levels of α-SMA, FAP, and type 1 collagen^38–41^, suggesting that, in our model, monocytes drive (myo)fibroblasts to adopt a disease-relevant phenotype. Notably, despite unchanged ACTA2 mRNA expression, immunohistochemistry revealed an increased proportion of (myo)fibroblasts strongly positive for α-SMA and FAP following co-culture with monocytes. This apparent discrepancy may, at least in part, be explained by the different time points analyzed, as ACTA2 expression was assessed after 24 h (h), whereas α-SMA protein accumulation was evaluated after 72 h. Therefore, it is plausible that transient or early transcriptional changes were not captured at the selected time point, while protein accumulation became evident at later stages.

Dermal fibroblasts isolated from SSc patients’ skin, when embedded in a 3D microenvironment such as biocompatible polyisocyanide (PIC-RGD) hydrogels, retain critical features of their in vivo behavior, including increased contraction, proliferation, and expression of α-SMA, collagen, and FAP. Inversely, under conventional 2D culture conditions, these cells rapidly lose their pro-fibrotic phenotype^42^. In this context, our results reinforce the importance of a 3D microenvironment not only in maintaining the pro-activated (myo)fibroblasts status induced by monocytes but also in enabling functional crosstalk between immune and stromal cells, which is largely absent or blunted in traditional 2D culture systems. Thus, the monocyte-induced upregulation of α-SMA and FAP reflects a biologically relevant activation of (myo)fibroblasts, occurring in a setting that more closely mimics the in vivo pro-inflammatory and pro-fibrotic niche.

Recently, the interaction between macrophages and fibroblasts in SSc has gained more attention. An elegant spatial multi-omics study integrating data from SSc patients and mice models identified the co-localization and signaling dynamics of fibroblasts and macrophages within fibrotic skin niches^9^. This disease-specific microenvironment enriched in both cell types was characterized by elevated TGF-β pathway activity and a strong correlation with patients’ clinical outcomes. The results suggest that fibrotic progression is an intricate process involving both the close spatial proximity of immune and stromal cells and dynamic signaling interactions occurring within organized niches. Sequential events, such as immune cell infiltration and disrupted cell communication, might progressively destabilize tissue homeostasis, promoting a shift toward a pro-fibrotic state^9^. In this context, our work adds a functional perspective by showing how infiltrating cells such as monocytes directly reshape (myo)fibroblast behavior, leading to increased contractile activity and a shift towards a contractile phenotype.

We identified that monocyte-driven (myo)fibroblast activation in the hydrogels involved signaling pathways that are critical in SSc, such as the IL-6/STAT3 and TGF-β/Smad pathways. In SSc patients’ skin, dermal fibroblasts and infiltrating mononuclear cells exhibit a prominent IL-6 expression. Additionally, elevated IL-6 levels are associated with more extensive skin involvement and reduced long-term survival^43^. Upon binding to its receptor (IL-6R), IL-6 can activate the JAK/STAT pathway, leading to STAT3 phosphorylation^44^. STAT3 is then translocated to the nucleus, binding to SIE, which consists of repeats of “IL-6 sis-inducible elements,” also known as STAT3 response elements, resulting in the expression of genes involved in cell proliferation, inflammatory response, and fibrogenesis^45,46^. Here, we demonstrated a clear phosphorylation of STAT3 in the hydrogels containing monocytes and (myo)fibroblasts. In addition, transcriptional activity of the SIE-reporter constructs significantly increased in the presence of monocytes, indicating that the JAK/STAT3 pathway plays a key role in monocyte-induced (myo)fibroblasts activation.

While IL 6 blocking therapies in SSc, such as tocilizumab, have demonstrated only modest effects in cutaneous fibrosis^47^, growing evidence suggests that targeting downstream signaling such as JAK/STAT3 could provide valuable clinical outcomes^48,49^. Therefore, our findings support a role for STAT3 in monocyte-induced (myo)fibroblast activation and strengthen the argument that targeting downstream pathways such as JAK/STAT3 may offer an effective approach in diseases like SSc with multifactorial cytokine involvement.

TGF-β/Smad signaling pathway is another important regulator of fibrosis, directly influencing the activation and differentiation of myofibroblasts^50^. Multiple abnormalities in the Smad signaling pathway have been detected in SSc^51^, including elevated phosphorylation of Smad2/3 in cultured SSc patients’ dermal fibroblasts^52,53^. In our study, we detected that monocytes led to Smad2 phosphorylation when co-cultured with (myo)fibroblasts in the hydrogels, further supporting the acquisition of an activated phenotype. However, unexpectedly, we observed decreased transcriptional activity of the SBE (Smad Binding Elements)-reporter constructs in the presence of monocytes, suggesting that some actions of TGF-β via Smad2/3 occur through distinct mechanisms. These differences in transcriptional activation mechanisms by Smad2 and Smad3^54^, as well as potential post-translational modifications^19^, could explain our contrasting results between increased Smad2 phosphorylation in the hydrogels and decreased transcriptional activity of the SBE-reporter constructs after co-culture with monocytes.

Building on these mechanistic insights, we also explored whether targeting the identified signaling pathways could modulate (myo)fibroblasts contractile activity in our 3D model. Treatment with SB-505,124, a TGF-β receptor type 1 inhibitor, at the beginning of the co-culture reduced drastically contraction on hydrogels containing (myo)fibroblasts and monocytes. In contrast, a later treatment (24 h after co-culture initiation) had no significant effect, suggesting that early activation of the TGF-β/Smad signaling pathway is a crucial step for contraction. SB-505,124 has previously been shown to reduce collagen deposition and myofibroblast numbers in an experimental model of fibrosis^55^, highlighting our 3D co-culture hydrogels as a relevant platform for testing therapies within this context. On the other hand, in an unexpected manner, immediate treatment of co-culture hydrogels with tofacitinib did not reverse (myo)fibroblasts contraction, however, it completely blocked this process when added later. Tofacitinib is a JAK1/JAK3 pathway inhibitor that has demonstrated potential efficacy in treating SSc patients, reducing skin thickness, and leading to positive impacts on musculoskeletal involvement^56^. Here, we believe that as co-culture progresses, pro-inflammatory cytokines, possibly secreted by monocytes and/or (myo)fibroblasts, could induce JAK/STAT pathway, explaining why tofacitinib had a stronger effect when added after one day of experimentation.

Having established that monocytes drive (myo)fibroblasts activation and contraction in our 3D skin model, we next investigated how monocytes themselves are modulated through interaction with (myo)fibroblasts, aiming to capture the bidirectional nature of these cells’ crosstalk. The co-culture of monocytes and (myo)fibroblasts in the hydrogels led to a distinct pattern of CD68 expression, with numerous CD68⁺ cells forming a surrounding layer covering the (myo)fibroblasts. This spatial arrangement bears some resemblances observed during wound healing process, where fibroblast-mediated signals guide macrophage positioning to support tissue repair^27^. Moreover, deformation fields generated by fibroblasts in fibrillar collagen matrices have been shown to dynamically attract macrophages over distances, coordinating their migration and activation^26^. Such similarities could indicate that our co-culture model recapitulates key aspects of the physiological crosstalk between (myo)fibroblasts and immune cells, supporting its biological relevance.

We further identified and classified the CD68^+^ cells into M1 or M2-like macrophages based on the expression of the scavenger receptor CD163. Flow cytometry analyses revealed a higher percentage of M2-like macrophages (CD45^+^CD68^+^CD163^+^) in the hydrogels following the co-culture with the (myo)fibroblasts. Consistent findings were reported in a human skin equivalent model, co-culturing SSc patients’ monocytes and fibroblasts, in which monocytes differentiated into CD163⁺ and CD206⁺ macrophages^57^. Here, many of the M2-like cells were found also expressing markers commonly associated with M1 macrophages, such as HLA-DR and CD86. Similarly, M1-like macrophages (CD45^+^CD68^+^CD163^−^) exhibited markers related to the M2 phenotype, such as CD206, indicating the occurrence of a mixed M1/M2 polarization phenotype. Such mixed phenotype was observed in circulating monocytes from SSc patients^58,59^ and has also been reported in other pathological contexts, including lung cancer^60^ and osteoarthritis^29^. Our findings suggest that cells’ interaction within the hydrogels might lead to the release of pro-inflammatory and pro-fibrotic mediators, fostering a spectrum of macrophage polarization state rather than a strict M1/M2 dichotomy.

Although M2 macrophages are widely associated with fibrosis, their isolated contribution may represent an oversimplified perspective^5^. In fact, a study using a murine model of SSc has shown that blocking M1 macrophage polarization can serve as an effective therapeutic strategy, particularly in the early stages of the disease^61^. These findings indicate a potential involvement of M1 pro-inflammatory macrophages in triggering or amplifying fibrotic responses, while M2-like macrophages contribute to sustaining and maintaining fibrosis over time. Here, when co-culturing (myo)fibroblasts with M1 or M2-like cells in the 3D collagen hydrogels, we detected strong spontaneous contraction of (myo)fibroblasts in the presence of both macrophage subtypes, an effect similarly observed using M1 and M2-like culture supernatant to stimulate hydrogels containing only (myo)fibroblasts, emphasizing that both M1- and M2-like macrophages contribute to (myo)fibroblast contractile activity. Additionally, the faster contraction observed in the co-culture condition (within 48 h), compared to delayed effects seen with supernatant exposure alone (after 96 days), points to a significant role for direct cell-cell contact in accelerating the fibrotic response^62^.

Differences in the expression of several macrophage markers have been described between GM-CSF- and M-CSF-derived macrophage populations, highlighting the influence of colony-stimulating factors on macrophage differentiation and phenotypic heterogeneity^63^. Accordingly, we acknowledge that our in vitro differentiation strategy may contribute to variability in macrophage phenotypes and should be considered for future studies.

Lastly, we pre-incubated the respective macrophage supernatants with neutralizing anti-TGF-β or anti-IL-6 antibodies to investigate the contribution of released soluble factors. We observed a marked reduction in (myo)fibroblast contractile activity when M2-like supernatants were treated with anti-TGF-β, whereas neutralizing IL-6 in M1-like supernatants did not yield the same effect. These results reinforce the crucial role of TGF-β in (myo)fibroblasts contraction and suggest that additional mediators released by M1-like macrophages, such as IL-1 or reactive oxygen species (ROS), might also contribute to the contraction process.

Despite providing valuable insights, our study has certain limitations. First, we acknowledge that the relatively low number of biological replicates in certain experiments represents a limitation that should be considered when interpreting the robustness of the findings. Second, incorporating only (myo)fibroblasts and monocytes/macrophages does not fully recapitulate the complexity of the pathological triad in SSc. Therefore, adding endothelial cells into 3D skin co-culture systems^64,65^ could provide a more comprehensive alternative for studying SSc-related vasculopathy, a crucial parameter in the disease pathogenesis and patients’ clinical outcomes^66^. Finally, the limited availability of isolated SSc patients’ cells (monocytes/macrophages and (myo)fibroblasts) prevented their inclusion in our study. Future research will focus on developing a personalized in vitro skin model by combining donor cells with experiments involving SSc patients’ samples.

## Methods

### Isolation and culture of primary skin dermal (myo)fibroblasts

Human primary dermal (myo)fibroblasts were obtained from punch skin biopsies (4 mm) of the dorsal mid-forearm of a healthy male donor under local asepsis and anesthesia. The collection and use of human skin samples were approved by the Medical Ethics Committee of Radboud University Medical Center (study number NL57997.091.16). All procedures were conducted in accordance with relevant guidelines and regulations. Written informed consent was obtained from the donor prior to sample collection. The material was placed in a 24-wells plate containing 2 ml DMEM Glutamax medium (Gibco, USA), supplemented with 100 U/ml penicillin, 100 mg/ml streptomycin, 100 mg/L pyruvate, and 20% fetal calf serum (FCS). Plates were incubated in appropriate culture conditions (37 °C, 5% CO_2_, 95% humidity) for 2 weeks until the spontaneous outgrowth of the primary (myo)fibroblasts. Every 3–4 days, the medium was partly refreshed. Subsequently, cells were cultured and expanded on plastic in T175 flasks in DMEM Glutamax medium supplied with 100 U/ml penicillin, 100 mg/ml streptomycin, 100 mg/L pyruvate, and 10% FCS. Cells were used between passages 5 and 12, with the majority of experiments performed at passages 5–9. Given the known phenotypic heterogeneity of primary skin fibroblast populations and the presence of cells with variable expression of myofibroblast markers (e.g., α-SMA), we used the terminology “(myo)fibroblasts” throughout the study.

### Human monocyte isolation and differentiation to M1 or M2-like macrophages

Monocytes were isolated from human peripheral blood mononuclear cells (PBMCs) obtained from buffy coats of healthy volunteers provided by the internal blood transfusion department (Sanquin, Nijmegen, The Netherlands). All volunteers gave written informed consent, and the study was conducted in accordance with institutional ethics committee (Commissie Mensgebonden Onderzoeksregio Arnhem-Nijmegen) approved protocols (project number: NVT 0397-02). PBMCs were obtained by Ficoll^®^-Paque PLUS (Sigma-Aldrich, USA) density centrifugation, following manufacturer’s guidelines. Monocytes were subsequently selected by magnetic-activated cell sorting (MACS) isolation, using CD14^+^ magnetic MicroBeads (130-050-201, Miltenyi Biotec, Germany) and MACS buffer: Phosphate Buffered Saline (PBS) with 1% FCS and 1.5% Anticoagulant Citrate Dextrose Solution (ACD)-A.

For macrophage differentiation, as previously described^29^, the obtained monocytes were seeded in 48-wells Upcell™ plates (Thermo Fisher Scientific, USA) at 250,000 cells/well and cultured in RPMI 1640 GlutaMAX™ medium (Gibco) supplemented with 10% FCS, 100 mg/L pyruvate, 100 U/mL penicillin, and 100 mg/mL streptomycin. Cells were differentiated during 6 days to M1-like macrophages using 50 ng/mL granulocyte-macrophage colony-stimulating factor (GM-CSF) (7954-GM-050/CF, R&D System, USA) or to M2-like macrophages using 20 ng/mL macrophage colony-stimulating factor (M-CSF) (216-MCC-025/CF, R&D Systems). On day 3 of culture, the medium with supplements was refreshed. Cells were activated on day 5, by adding 20 ng/mL interferon (IFN)-γ (570202, BioLegend, USA) or 10 ng/mL interleukin (IL)-4 (204-IL, R&D Systems) for the M1 or M2-like macrophages, respectively. The supernatant was collected on the last day of culture (day 6), spun at 400×g for 10 min to remove cell debris, and stored at -80 °C until use. Finally, cells were detached and collected by 1-hour incubation in PBS at 4 °C.

### 3D skin co-culture of monocytes/macrophages and (myo)fibroblasts in a collagen hydrogel contraction model

Primary skin dermal (myo)fibroblasts were washed with saline and detached from the culture flasks using trypsin (T1426-100MG, Sigma-Aldrich, USA) at 37 °C. Subsequently, 10 ml of DMEM medium with 10% FCS was added to inactivate the trypsin. PBMCs, isolated monocytes (CD14^+^), PBMCs without monocytes (depleted from CD14^+^ cells by MACS), and M1 or M2-like macrophages were obtained as previously described. To make each 3D collagen hydrogel^15^, 20 µl 10 x Minimum Essential Medium Eagle (Cat. No. M0275, Sigma-Aldrich), 10 µl Sodium Bicarbonate 7.5% solution (Gibco), 150 µL Type 1 Bovine Collagen Solution (#5005, PureCol^®^, Advanced BioMatrix, Sweden), and 90 µl cell suspension were sequentially mixed and gently homogenized.

Hydrogels contained either 2*10^5^ (myo)fibroblasts alone or a mix of 2*10^5^ (myo)fibroblasts with 1*10^6^ PBMCs, monocytes, or PBMCs depleted from CD14^+^ cells. The hydrogels containing M1 or M2-like macrophages had 5*10^5^ cells together with 2*10^5^ (myo)fibroblasts. Of the collagen-cell mixture, 250 µl was used per well in 48-well plates. Hydrogels were incubated under standard culture conditions (37 °C, 5% CO_2_) until solidification for at least 1 h. Finally, 750 µl of RPMI 1640 GlutaMAX™ (Gibco) supplemented with 100 U/ml penicillin, 100 mg/ml streptomycin, 100 mg/L pyruvate, and 10% Human Pooled Serum (HPS) were added, and the hydrogels were put back in the culture incubator.

For pharmacological inhibition of signaling pathways, hydrogels were treated with Tofacitinib (1 µM) (LC Laboratories, USA), SB-505,124 (5 µM) (Sigma-Aldrich), IKK-16 (1,5 mM) (MedChemExpress, USA), or TAK-242 (1 mM) (R&D Systems), diluted into culture medium and subsequently added to the hydrogels after solidification. In another experimental setting, hydrogels containing only (myo)fibroblasts were exposed to M1 or M2-like macrophage culture supernatant. For this, after hydrogels’ solidification, 250 µL of M1 or M2-like culture supernatant was added to each well. One day after (24 h), 500 µL RPMI medium was added. To analyze the role of soluble mediators possibly released by M1 or M2-like macrophages in (myo)fibroblasts contraction, anti-IL-6 (Cat. No. 501101, BioLegend, USA) (1 µg/ml) and anti-TGF-β (#MAB1835, R&D Systems) (100 ng/ml) neutralizing antibodies were used. An IgG isotype (Cat. No. 401501, BioLegend) antibody was used as a control. M1 or M2-like macrophage supernatant was incubated for 1 h with the respective neutralizing antibodies and then added to the hydrogels.

In all experiments, three technical replicates were used. Spontaneous contraction of the hydrogel was macroscopically evaluated over time by scanning plates on a standard office flatbed scanner with 600 dpi resolution. The hydrogels were not detached from the wells after solidification, remaining adhered to the culture surface throughout the experiments; consequently, contraction occurred under mechanically constrained conditions imposed by surface adhesion. Hydrogel contraction was quantified by measuring the area of the hydrogels using Fiji ImageJ software and calculated as follows:

$$\:\mathrm{C}\mathrm{o}\mathrm{n}\mathrm{t}\mathrm{r}\mathrm{a}\mathrm{c}\mathrm{t}\mathrm{i}\mathrm{o}\mathrm{n}\:=\left(\frac{\mathrm{A}\mathrm{r}\mathrm{e}\mathrm{a}\:\mathrm{o}\mathrm{f}\:\mathrm{u}\mathrm{n}\mathrm{c}\mathrm{o}\mathrm{n}\mathrm{t}\mathrm{r}\mathrm{a}\mathrm{c}\mathrm{t}\mathrm{e}\mathrm{d}\:\mathrm{p}\mathrm{l}\mathrm{u}\mathrm{g}-\mathrm{A}\mathrm{r}\mathrm{e}\mathrm{a}\:\mathrm{o}\mathrm{f}\:\mathrm{c}\mathrm{o}\mathrm{n}\mathrm{t}\mathrm{r}\mathrm{a}\mathrm{c}\mathrm{t}\mathrm{e}\mathrm{d}\:\mathrm{p}\mathrm{l}\mathrm{u}\mathrm{g}}{\mathrm{A}\mathrm{r}\mathrm{e}\mathrm{a}\:\mathrm{o}\mathrm{f}\:\mathrm{u}\mathrm{n}\mathrm{c}\mathrm{o}\mathrm{n}\mathrm{t}\mathrm{r}\mathrm{a}\mathrm{c}\mathrm{t}\mathrm{e}\mathrm{d}\:\mathrm{p}\mathrm{l}\mathrm{u}\mathrm{g}}\right)\times\:100\mathrm{\%}$$

### Flow cytometry

Collagen hydrogels were enzymatically digested (for 1 h on a roller at 37 °C) using a mixture of collagenase D (1:50) (Roche, Switzerland) and DNAse (1:100) (Roche) diluted in PBS. Reaction was stopped by adding cold PBS. The hydrogels were mechanically disrupted by gently pipetting up and down, then centrifuged at 300 × g for 5 min. After centrifugation, supernatant was discarded and the obtained single-cell suspension was washed twice with PBS, followed by isolation of the immune cell fraction using CD45^+^ magnetic MicroBeads (130-045-801, Miltenyi Biotec). The isolated CD45^+^ portion was used to access cells’ phenotype by flow cytometry.

Briefly, leukocytes isolated from the digested hydrogels were first stained for cell viability with ViaKrome 808 (1.5:1000 in PBS) (Beckman Coulter, USA) for 30 min at 4 °C in the dark. Then, samples were washed and incubated for 20 min at room temperature (protected from light) with fluorescently labeled extracellular antibodies. Subsequently, intracellular staining was performed using the Cyto-Fast™ Fix/Perm Buffer Set (BioLegend) following the manufacturer’s instructions. Cells were acquired in a Cytoflex LX 21-color flow cytometer (Beckman Coulter). Data analyses were conducted using Kaluza software version 2.1.3 (Beckman Coulter). A list of all extra- and intracellular antibodies used is provided in **Supplementary Table 1**.

### Immunohistochemistry

Collagen hydrogels were collected and fixed in a 10% buffered formalin solution, followed by embedding in paraffin. Five-micrometer (µm) thick paraffin sections were cut from formalin-fixed blocks and added to glass slides. Identified samples were then deparaffinized with xylol wash and rehydrated with ethanol. Afterward, the material passed by an antigen retrieval process using 10 mM citrate (pH 6.0) at room temperature. Endogenous peroxidase activity was inhibited by incubating the samples with 3% H_2_O_2_ in PBS for 15 min, and subsequently, non-specific binding was blocked using 20% Normal Goat Serum (NGS)/PBS or horse serum. Sections were then incubated with primary rabbit -anti-human alpha-smooth muscle actin (α-SMA) (1:200) (ab5694, Abcam, UK), - fibroblast activation protein (FAP) (1:100) (ab207178, Abcam), - Phospho-SMAD2 (1:100) (#3108L, CellSignaling, USA), - Phospho-Stat3 (1:50) (#9145, CellSignaling), or mouse anti-human - CD68 (#MCA1815, BioRad, USA) (1:200) antibodies, for 1 hour at room temperature. Next, tissues were incubated with corresponding secondary antibodies: goat anti-rabbit biotinylated IgG antibody (1:400) (#BA-1000, VectorLabs, USA) or horse anti-mouse biotinylated IgG antibody (1:200) (#BA-2001, VectorLabs) and then labeled with the avidin-biotin complex (1:100) (VECTASTAIN, Thermo-Fisher). Finally, the reaction was revealed using 3,3’-diaminobenzidine (bright DAB, Immunologic). All slides were counterstained with hematoxylin and mounted with a cover slip (Permount, Thermo-Fischer, USA). All images were acquired using a Leitz DMRD microscope (D-35578 Wetzlar, Leica Microsystems, Mannheim, Germany) equipped with a digital camera system for image acquisition.

### RNA isolation and quantitative real-time PCR

Total RNA was extracted from the CD45^−^ cell fraction, (myo)fibroblasts, using the RNeasy Mini Kit (Qiagen, Germany), following the manufacturer’s instructions. RNA concentrations were quantified with a Nanodrop photo spectrometer (Thermo Scientific, USA), and any residual DNA was removed by treating samples with DNase I (Life Technologies, USA). Subsequently, RNA was reverse-transcribed into cDNA in a single-step reverse transcription PCR, using oligo dT primer and 200U M-MLV Reverse transcriptase (All Life Technologies, USA), in a thermocycler. Gene expression was evaluated on the produced cDNA samples using SYBR Green Master Mix (Applied Biosystems, USA) and 0.2 mM of validated specific primers (Biolegio, Nijmegen, the Netherlands) in a quantitative real-time polymerase chain reaction (qPCR). Primers sequences are provided in **Supplementary Table 2**. All reactions were carried out in triplicate, using a StepOnePlus Real-Time PCR System (Applied Biosystems). Reference genes used were GAPDH and RPS27A. The relative gene expression (2^−ΔΔCt^) was calculated using the average values of the reference genes.

### Reporter luciferase assay

Reporter constructs cloned in the same human primary skin dermal (myo)fibroblasts were obtained from (18). Reporter assays, such as Sis-inducible element (SIE), a STAT-responsive regulatory element; SMAD binding element (SBE); cyclic AMP response element (CRE); nuclear factor of activated T-cells 5 (NFAT-5); interferon-stimulated response element (ISRE), and nuclear factor-κB response element (NF-κB), for signaling pathways known to be active in fibroblasts, were tested (**Supplementary Table 3**). Hydrogels containing monocytes and the different (myo)fibroblasts strains were prepared using the previously mentioned cell ratio (5:1). The co-culture was incubated for 24 h and then cells were lysed using Cell Culture Lysis Reagent (Promega, USA). Next, Nano-Glo luciferase reagent (Promega) was added according to the manufacturer’s protocol. Fluorescence was measured at 590 nm with a Clariostar (BMG Labtech, Germany). An appropriate positive control, known to activate each (myo)fibroblasts transfected with transcription factor-responsive reporter construct, was used.

### Statistical analysis

Statistical analyses were performed with GraphPad Prism 10 (10.0.3 version; GraphPad Software Inc., USA). Depending on data normality, either Student’s t-test, Wilcoxon test, one-way ANOVA, or Kruskal–Wallis test was performed. The specific statistical test used for each analysis is indicated in the respective figure legends. Differences were considered significant with a p-value of less than 5% (*p* < 0.05).

## Supplementary Information

Below is the link to the electronic supplementary material.

Supplementary Material 1

## Data Availability

All data generated or analyzed during this study are included in this published article (and its Supplementary Information files).
