# Supplementary material for: Monocytes strongly induce (myo)fibroblast contraction in a 3D skin model to understand inflammation-fibrosis crosstalk
Source: Sci Rep. 2026 Jul 19;16:28481. doi: 10.1038/s41598-026-62996-0 (PMC13569832; doi:10.1038/s41598-026-62996-0)
Supplement: Supplementary file 1 [file 41598_2026_62996_MOESM1_ESM.pdf]

SUPPLEMENTAL FIGURES

Supplemental Figure 1

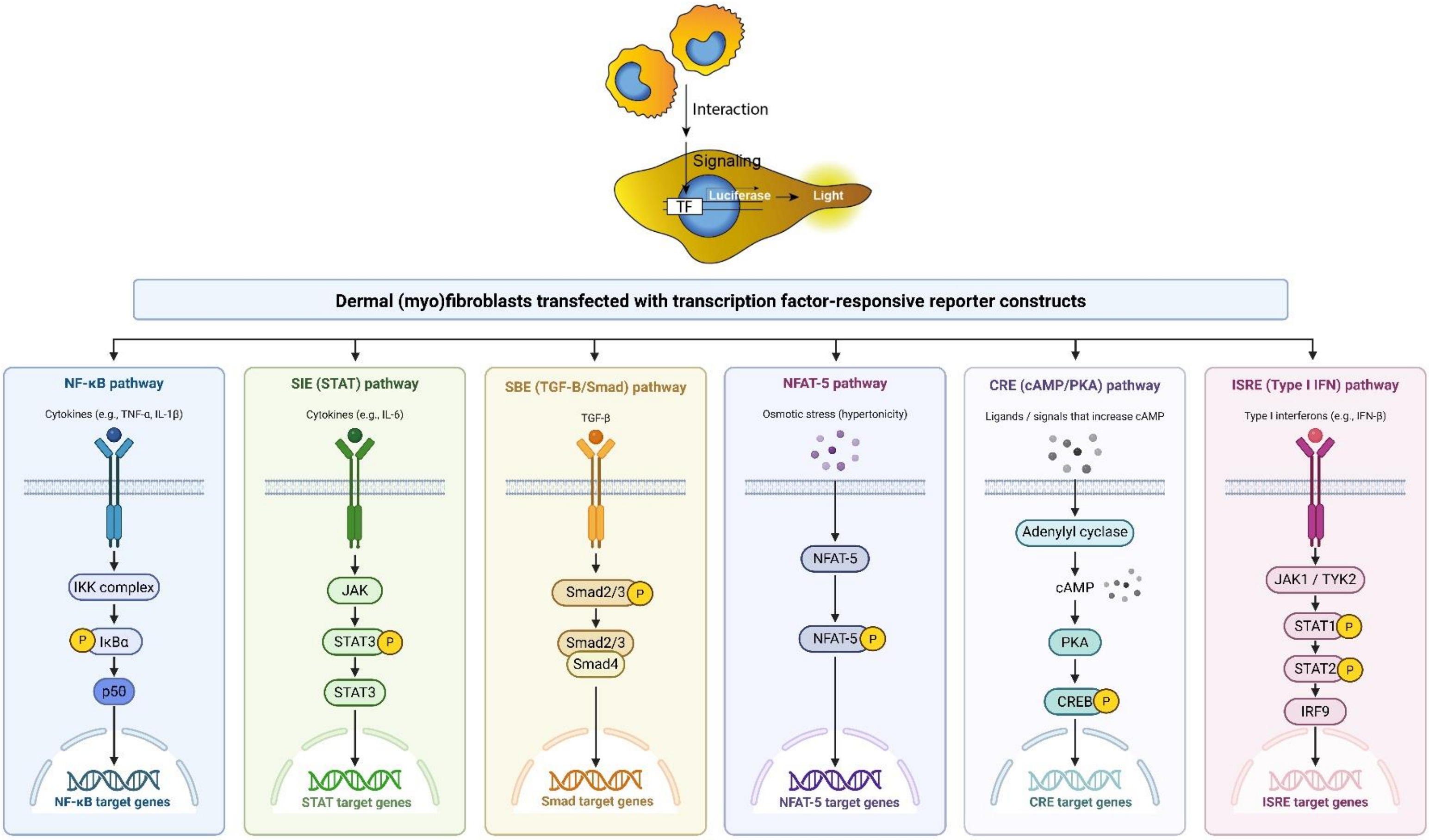

Supplementary Figure 1. Schematic representation of the dermal (myo)fibroblasts transfected with transcription factor-responsive reporter constructs. Upon stimulation (e.g., co-culture with monocytes/macrophages), transcriptional activation drives luciferase expression, which can be subsequently quantified by luminescence as an indirect measure of pathway activity. The cells were used to elucidate the mechanisms underlying (myo)fibroblasts activation triggered in response to monocytes/macrophages. NF-κB response elements represent classical inflammatory signaling; STAT-inducible elements (SIE) reflect JAK/STAT activation; Smad-binding elements (SBE) capture canonical TGF-β signaling; Nuclear factor of activated T-cells 5 (NFAT-5) response elements are associated with osmotic stress signaling; Cyclic adenosine monophosphate (cAMP) response elements (CRE) reflect cAMP/PKA pathway activation; Interferon-stimulated response elements (ISRE) are indicative of type I interferon responses.

Supplemental Figure 2

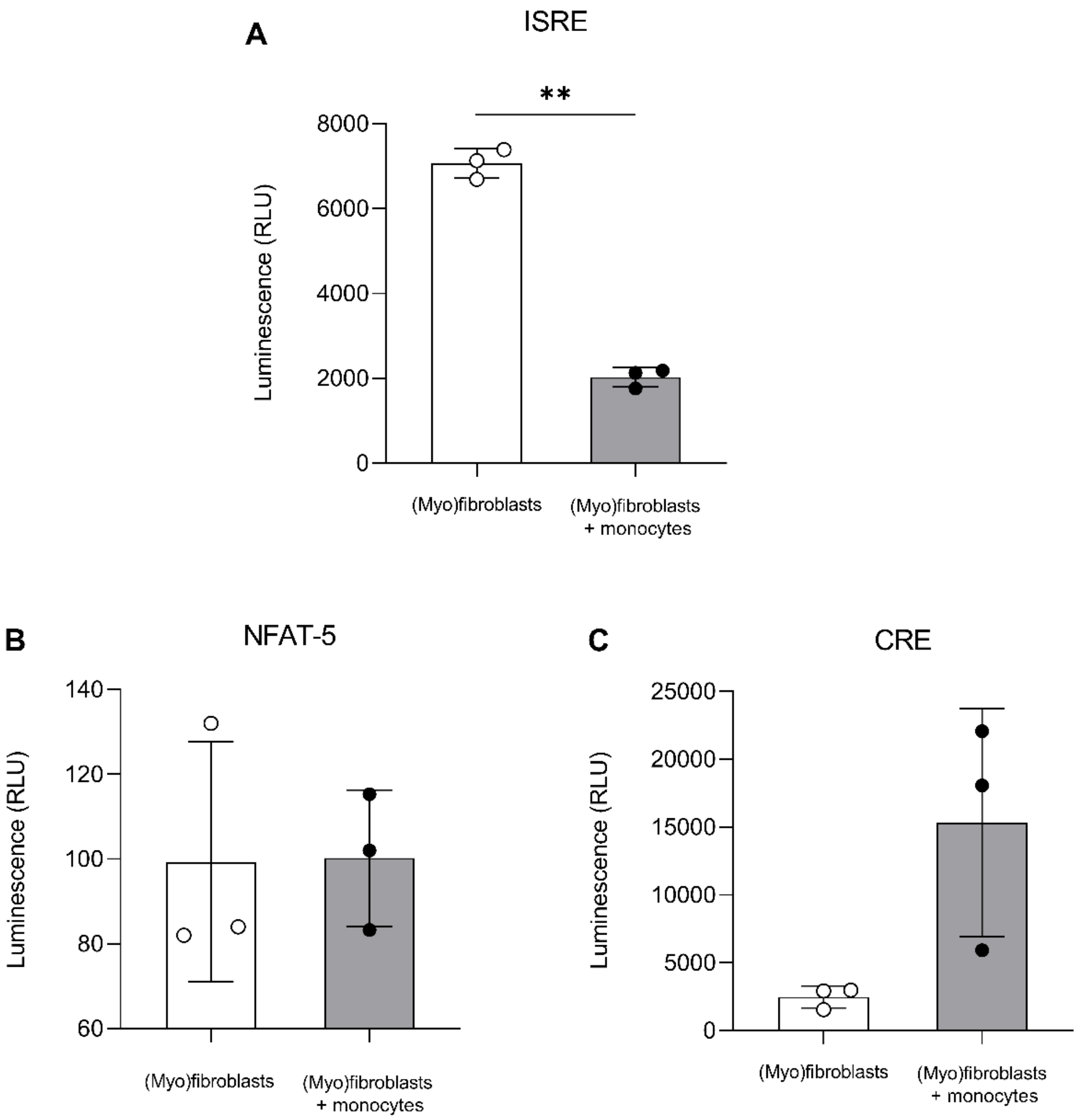

Supplemental Figure 2. Additional dermal (myo)fibroblasts transfected with transcription factor-responsive reporter constructs tested in co-culture with monocytes. Dermal (myo)fibroblasts transfected with transcription factor-responsive reporter constructs ISRE (**A**), NFAT-5 (**B**), or CRE (**C**) were co-cultured with monocytes in collagen hydrogels for 24 hours. Luminescence was measured as a readout of transcriptional activity. (Myo)fibroblast-only hydrogels were included as controls. Each dot represents the mean of three technical replicates from one PBMC donor (n = 3). Statistical analysis was performed using Student's t-test (**p < 0.01). ISRE: Interferon-stimulated response element (ISRE); NFAT- 5: Nuclear factor of activated T-cells 5, and CRE: Cyclic AMP response element.

Supplemental Figure 3

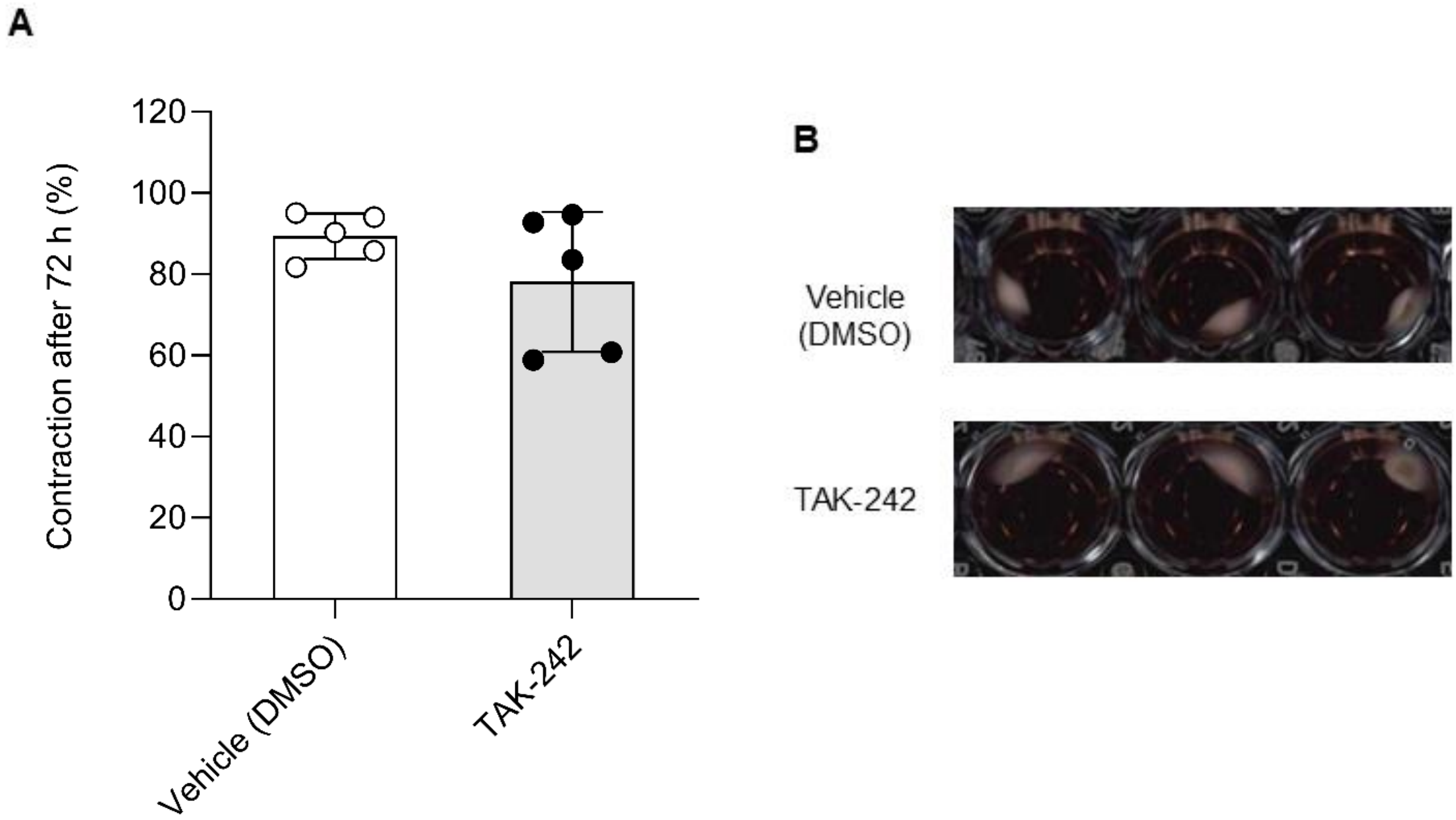

Supplementary Figure 2. Blocking the TLR4 signaling does not affect (myo)fibroblasts contraction. Co-cultured collagen hydrogels were treated with TK-242 (1 µM) or vehicle (DMSO), and contraction was measured after 72 hours. (**A**) Quantification of hydrogel contraction showing no significant differences between control and TAK-242–treated conditions, indicating that TLR-4 inhibition had no impact to halt myofibroblasts contraction. Each symbol represents the mean of three technical replicates from one PBMC donor (n = 5). Statistical analysis was performed using Student's t-test. (**B**) Representative scanned images of co-cultured hydrogels treated with TAK-242.

Supplemental Figure 4

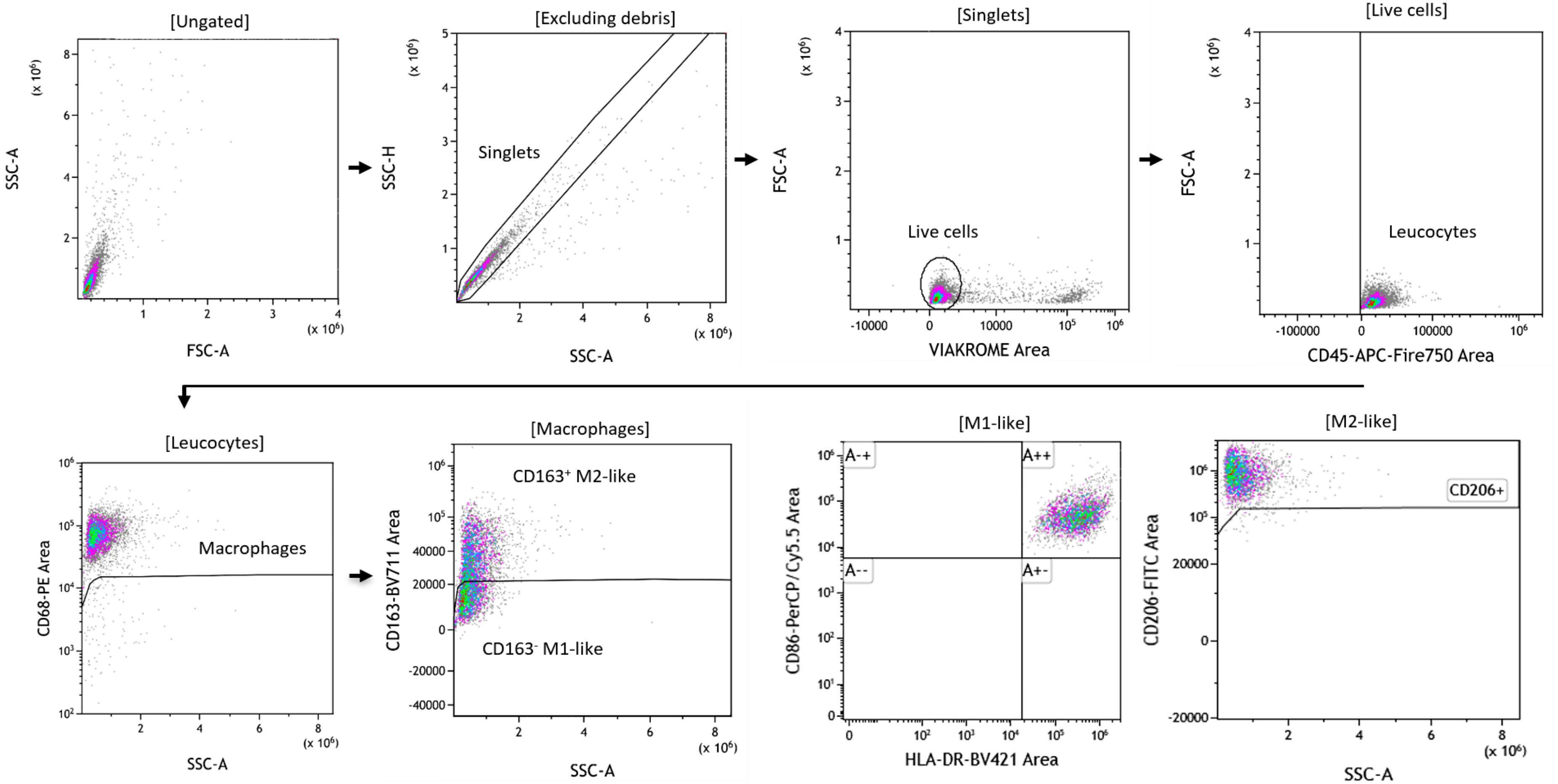

Supplemental Figure 4. Flow cytometry gating strategy for monocytes-containing hydrogel plugs. Collagen hydrogels co-cultured with (myo)fibroblasts and monocytes were enzymatically digested to obtain a single-cell solution, followed by MACS-CD45$^+$ isolation and flow cytometric analyses. Gating was performed sequentially on single, live cells, total leukocytes (CD45$^+$). Within the leukocyte population, macrophages were identified as CD45$^+$CD68$^+$ cells. Macrophages were further subdivided into M1-like (CD163$^-$HLA-DR$^+$CD86$^+$) and M2-like (CD163$^+$CD206$^+$) subsets.

Supplemental Figure 5

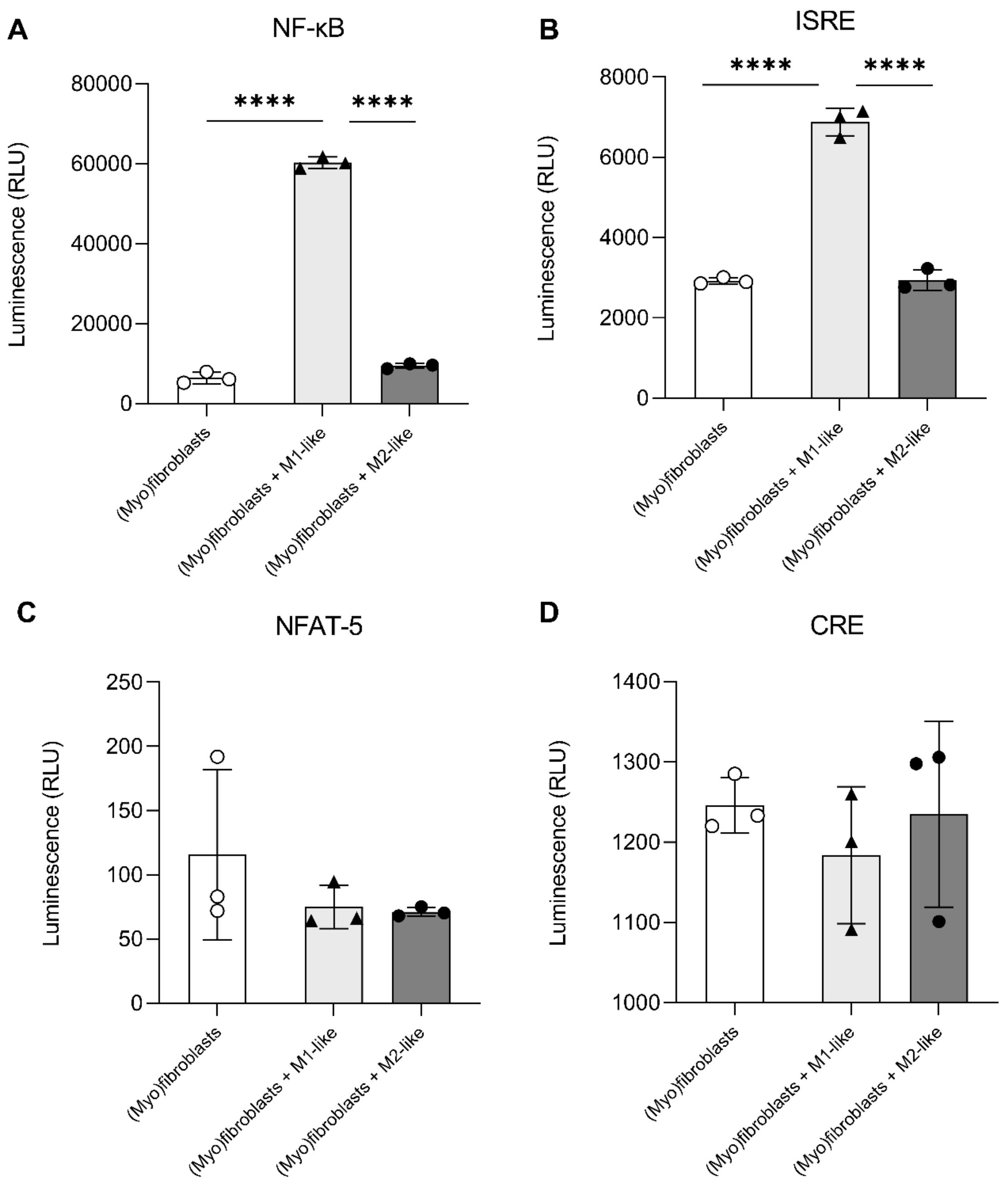

Supplemental Figure 5. (Myo)fibroblast transfected with transcription factor-responsive reporter constructs co-cultured with M1- or M2-like macrophages. (Myo)fibroblasts transfected with transcription factor-responsive reporter constructs NF-κB (**A**), ISRE (**B**), NFAT-5 (**C**), or CRE (**D**) were co-cultured with either M1- or M2-like macrophages, and luciferase activity was measured for respective signaling reporters. (Myo)fibroblast-only hydrogels were included as controls. Each shape represents the average of 3 technical replicates of 1 PBMC donor, n = 3. Statistical analyses were performed using Student's t-test (****$p<0.0001$). NF-κB: Nuclear factor-κB response element; ISRE: Interferon-stimulated response element (ISRE); NFAT-5: Nuclear factor of activated T-cells 5, and CRE: Cyclic AMP response element.

Supplementary Table 1: List of antibodies used for extra- and intracellular staining

Antigen	Clone	Dilution	Fluorochrome	Supplier
CD45	HI30	1:100	APC-Fire 750	BioLegend
CD86	BU63	1:200	PerCP/Cy5.5	BioLegend
HLA-DR	L243	1:50	BV421	BioLegend
CD163	GH1/61	1:200	BV711	BioLegend
CD206	15-2	1:200	FITC	BioLegend
CD68*	Y1/82A	1:100	PE	BioLegend

*CD68 was used for only intracellular staining

Supplementary Table 2: List of primer sequences used for qPCR

Gene	Forward primer 5' – 3'	Reverse primer 5' – 3'
GAPDH	ATCTTCTTTTGCGTCGCCAG	TTCCCCATGGTGTCTGAGC
RPS27A	TGGCTGTCCTGAAATATTATAAGGT	CCCCAGCACCACATTCATCA
PDPN	GGTGCAATCATCGTTGTGGTTA	TTCAGCTCTTTAGGGCGAGTAC
COL1A1	AGATCGAGAACATCCGGAG	AGTACTCTCCACTCTTCCAG
IL6	AGCCCACCGGGAACGA	GGACCGAAGGCGCTTGT
FN1 EDA	TTCAGACTGCAGTAACCAACAT	GGTCACCCTGTACCTGGAAAC
COL3A1	CCTGGAATCTGTGAATCATGCC	TGCGAGTCCTCCTACTGCTA
ACTA2	CTGACCCTGAAGTACCCGATA	GAGTGGTGCCAGATCTTTTCC
HLA-DR	CCCTGCAGCACCACAAC	GGAACCACCTGACTTCAATGC
PLOD2	AAGACTCCCCTACTCCGGAAA	AGCAGTGGATAATAGCCTTCCAA

Supplementary Table 3: Reporter assays for transcription factor (TF)/signaling pathways known to be active in fibroblasts

Pathway	TF(s)	TF Element (Abbreviation Construct)	Positive Control	Reference
NFκB	NFκB:p65	Nuclear factor κ B response element (NFκB)	IL-1β (1 ng/mL)	OHMORI; HAMILTON, 1995
cAMP/PKA	CREB	Cyclic AMP response element (CRE)	Forskolin (10 µM)	TAN et al., 1994
TGF-β	SMAD3:SMAD4	SMAD binding element (SBE)	TGF-β1 (1 ng/mL)	REISDORF et al., 2001
INF-α	STAT1:STAT2	Interferon-stimulated response element (ISRE)	IFN-α (100 ng/mL)	OHMORI; HAMILTON, 1995
IL-6	STAT3:STAT3	Sis-inducible element (SIE)	IL-6 (10 ng/mL)	CHAKRABORTY et al., 2017
Calcium/calcineurin: hyperosmotic signaling	NFAT5	Nuclear factor of activated T-cells 5	+100 milliosmole	HERBELET et al., 2018
